# Supplementary material for: A Compact Real-Time PCR System for Point-of-Care Detection Using a PCB-Based Disposable Chip and Open-Platform CMOS Camera
Source: Sensors (Basel). 2025 May 17;25(10):3159. doi: 10.3390/s25103159 (PMC12115960; doi:10.3390/s25103159)
Supplement: Supplementary file 1 [file sensors-25-03159-s001.zip › Supplementary File S1 – System Schematic and Module Connections.pdf]

Sheet: Hub
File: Hub.sch

Sheet: MCU_PIC
File: MCU_PIC.sch

Sheet: PCR
File: PCR.sch

Sheet: /		
File: CamPCR.sch		
**Title:**		
Size: A4	Date:	**Rev:**
KiCad E.D.A. kicad (5.1.9)-1		Id: 1/4

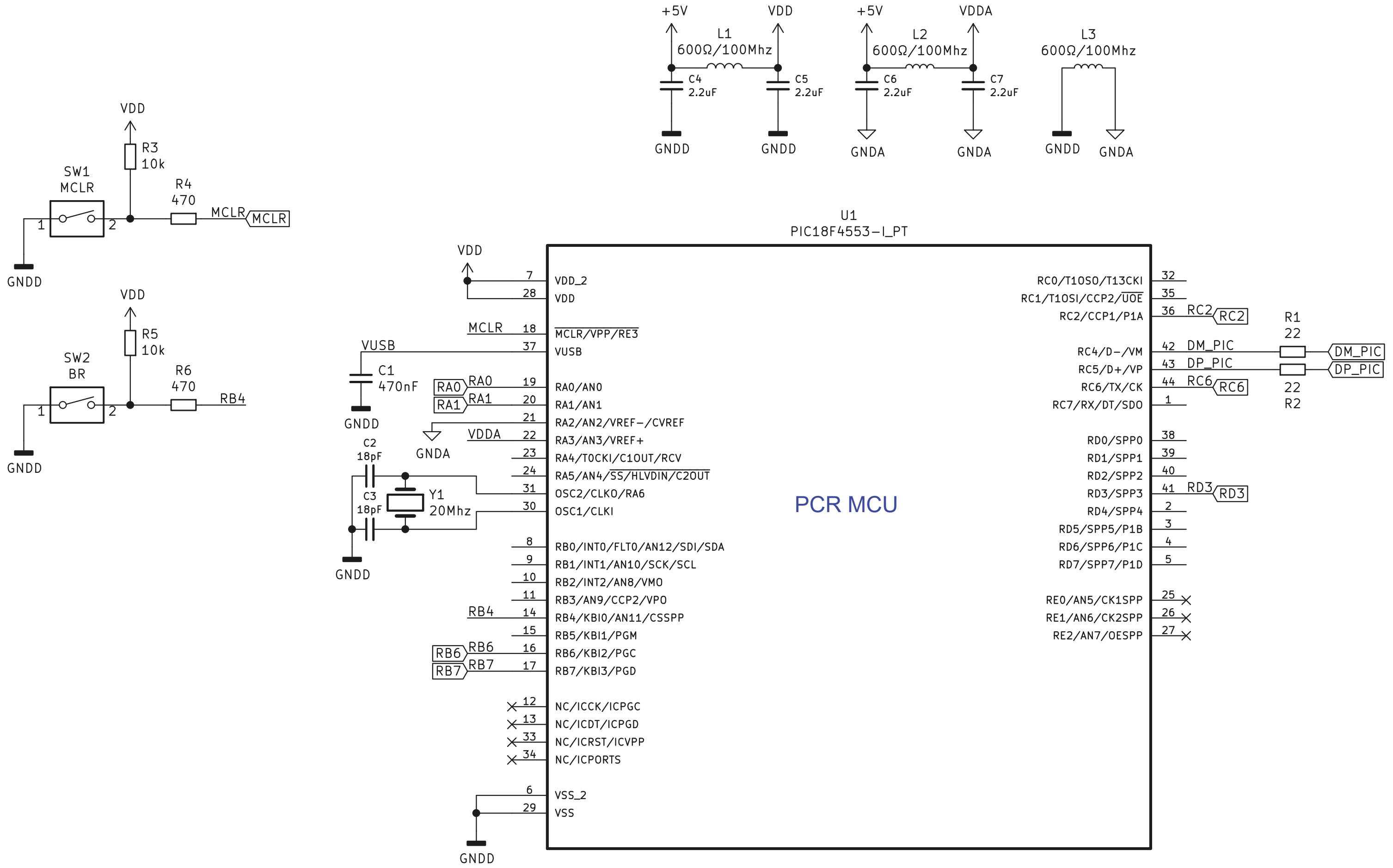

PCR MCU (PIC 18F4553)

Sheet: /MCU_PIC/
File: MCU_PIC.sch

Title:

Size: A4 | Date: | **Rev:**

KiCad E.D.A. kicad (5.1.9)-1 | Id: 2/4

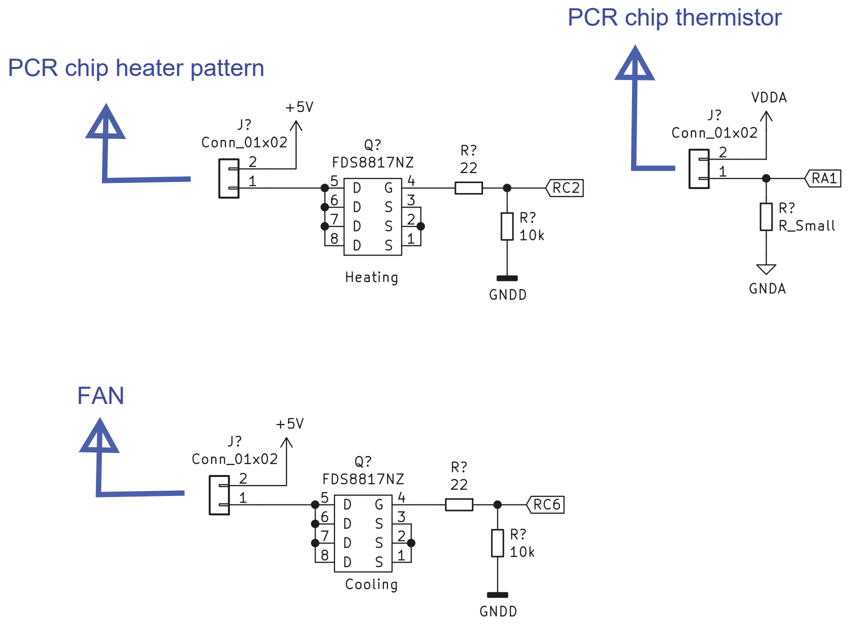

PCR chip and FAN driving FET's

Sheet: /PCR/
File: PCR.sch

Title:

Size: A4	Date:	**Rev:**
KiCad E.D.A. kicad (5.1.9)-1		Id: 3/4

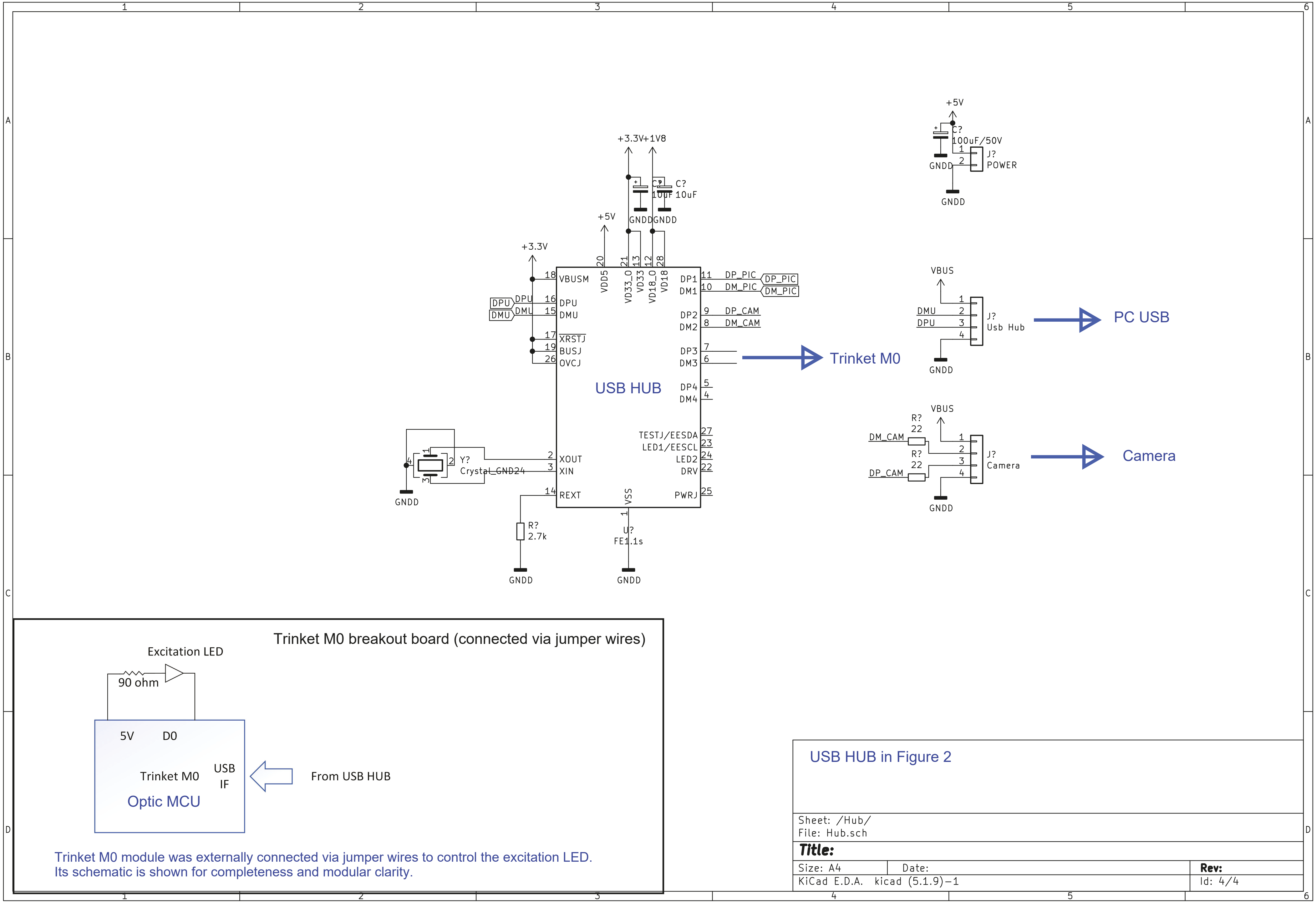

+3.3V
+1V8
+5V
C?
10uF
GNDD
VBUSM
DPU
DMU
XRSTJ
BUSJ
OVCJ
VDD5
VD33_O
VD33
VD18_O
VD18
DP1
DM1
DP2
DM2
DP3
DM3
DP4
DM4
DP_PIC
DM_PIC
DP_CAM
DM_CAM
USB HUB
Trinket M0
TESTJ/EESDA
LED1/EESCL
LED2
DRV
PWRJ
XOUT
XIN
REXT
VSS
Y?
Crystal_GND24
R?
2.7k
U?
FE1.1s
100uF/50V
J?
POWER
VBUS
Usb Hub
PC USB
22
Camera
Trinket M0 breakout board (connected via jumper wires)
Excitation LED
90 ohm
5V
D0
USB
IF
Optic MCU
From USB HUB
Trinket M0 module was externally connected via jumper wires to control the excitation LED.
Its schematic is shown for completeness and modular clarity.
USB HUB in Figure 2
Sheet: /Hub/
File: Hub.sch
Title:
Size: A4
Date:
KiCad E.D.A. kicad (5.1.9)-1
Rev:
Id: 4/4
