# Supplementary material for: A Compact Real-Time PCR System for Point-of-Care Detection Using a PCB-Based Disposable Chip and Open-Platform CMOS Camera
Source: Sensors (Basel). 2025 May 17;25(10):3159. doi: 10.3390/s25103159 (PMC12115960; doi:10.3390/s25103159)
Supplement: Supplementary file 1 [file sensors-25-03159-s001.zip › Supplementary_Materials.pdf]

Supplementary Materials for

A Compact Real-Time PCR System for Point-of-Care Detection Using a PCB-Based Disposable Chip and Open-Platform CMOS Camera

Figure S1. Structure and assembly order of the PCB-based PCR chip.

(a) Front and back views of the PCB substrate (200 μm thick). The back side includes the heating pattern and an NTC thermistor, with a magnified view highlighting their layout and positions.

(b) Front and back views of the molded polycarbonate chamber. The uncoated region, approximately 14 mm × 15 mm, indicates the chamber opening and is provided for reference. The chamber was molded into a 2.4 mm-thick polycarbonate part, with a groove depth of 1.5 mm. The exact groove width is not explicitly marked but can be inferred from the uncoated region.

(c) Front and back views of the plastic housing.

(d) Exploded view illustrating the sequential assembly using single-sided (1510) and double-sided (9795R) medical-grade adhesive tapes, along with silicone caps for inlet and outlet sealing.

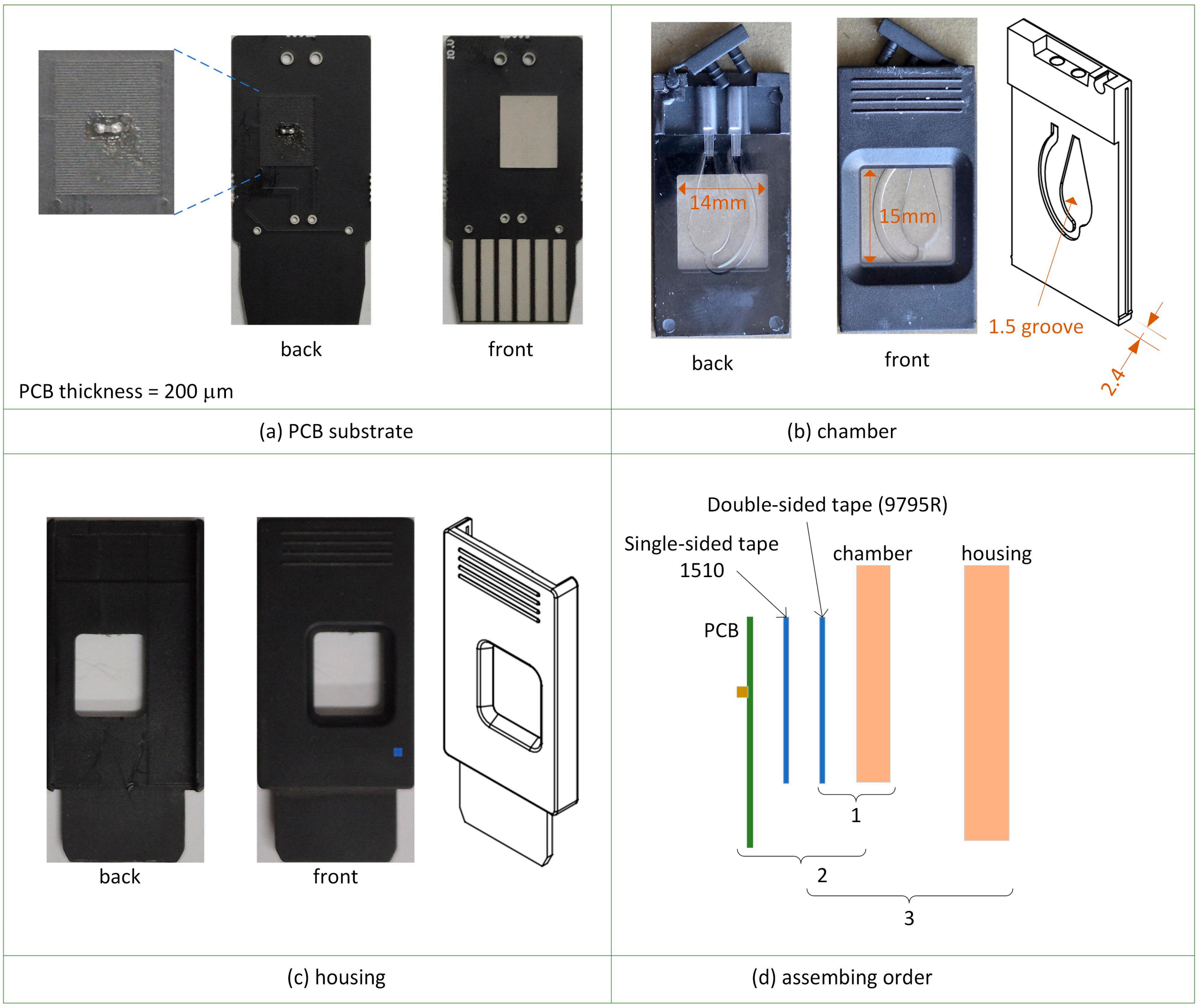

Figure S2. Host-local architecture for PCR thermal cycling.

The host PC handles high-level user interactions, protocol management, and state monitoring through file-based communication (WriteFile/ReadFile). The local system (microcontroller) runs a periodic control loop that continuously receives commands from the

host and updates the system state based on sensor and actuator feedback. This architecture separates latency-tolerant user operations from time-sensitive hardware control, ensuring real-time performance with minimal software complexity.

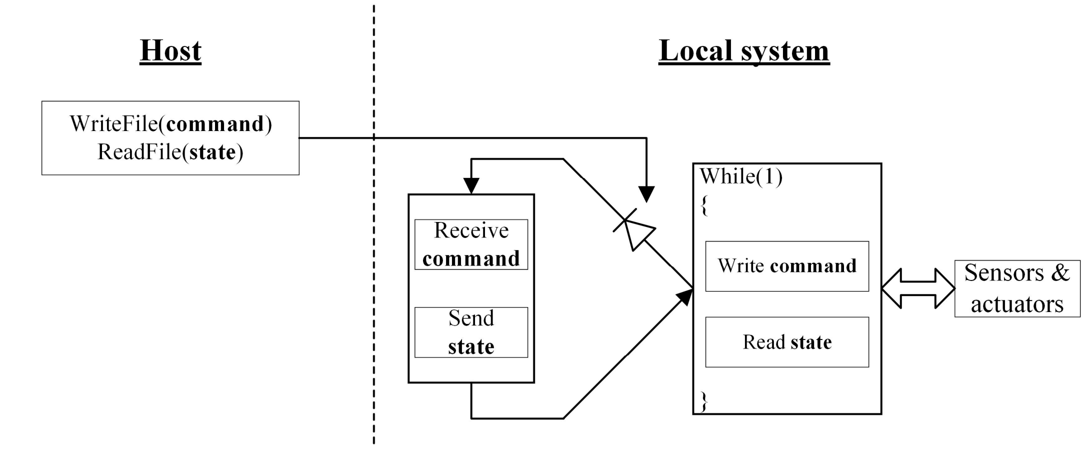

Figure S3. Host-local architecture for PCR thermal cycling.

The Optic Server includes Shot Thread, Camera Thread, and Optic Thread. During a SHOT request, the Shot Thread initiates LED activation and image capture, which are processed and normalized before returning RFU to the PCR Controller.

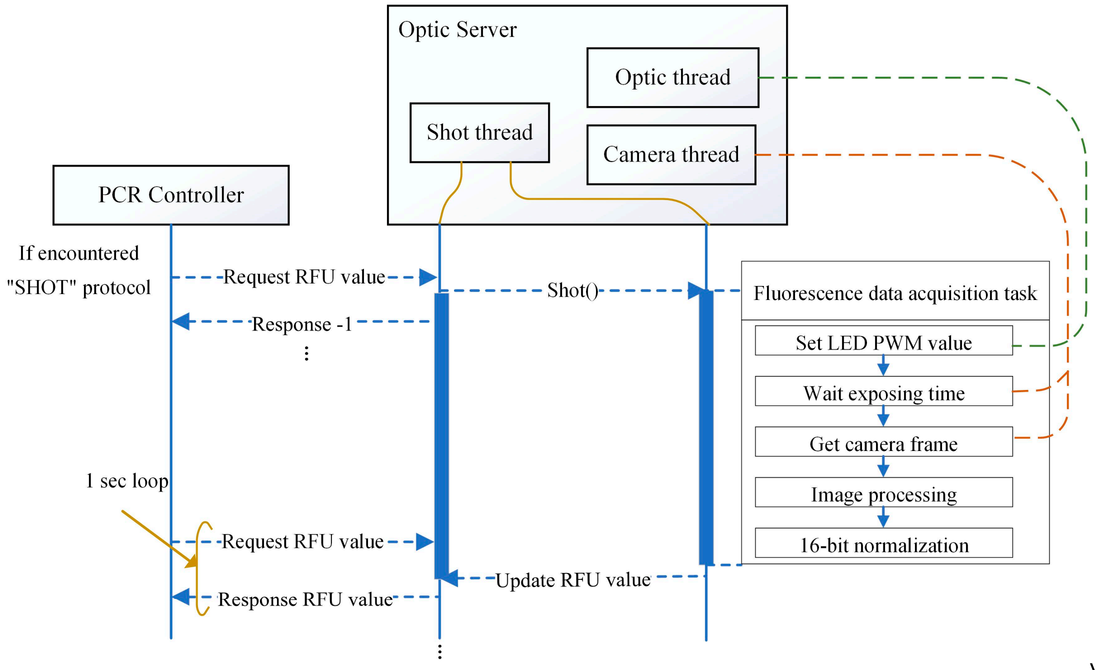

\

Equation S1. PID control law used in the proposed PCR system.

The control signal u is derived from the error between the measured temperature T and the target protocol temperature $A(i).T$. PWM control is updated every 2 ms based on filtered temperature input, with K_p, K_i, K_d tuned experimentally for thermal stability.

$$u = K_p e + K_i \int e dt + K_d \frac{de}{dt}, \quad e = T - A(i).T$$
